# Prognostic and predictive factors for the efficacy and safety of trastuzumab deruxtecan in HER2-positive gastric or gastroesophageal junction cancer

**DOI:** 10.1007/s10120-024-01560-z

**Published:** 2024-11-02

**Authors:** Amane Jubashi, Izuma Nakayama, Shigehiro Koganemaru, Naoya Sakamoto, Shioto Oda, Yuki Matsubara, Yu Miyashita, Seiya Sato, Shinpei Ushiyama, Akinori Kobayashi, Ukyo Okazaki, Dai Okemoto, Kazumasa Yamamoto, Saori Mishima, Daisuke Kotani, Akihito Kawazoe, Tadayoshi Hashimoto, Yoshiaki Nakamura, Yasutoshi Kuboki, Hideaki Bando, Takashi Kojima, Takayuki Yoshino, Hisamitsu Miyaaki, Kazuhiko Nakao, Kohei Shitara

**Affiliations:** 1 Department of Gastroenterology and Gastrointestinal Oncology, National Cancer Center Hospital East, 6-5-1 Kashiwanoha, Kashiwa, Chiba 277-8577 Japan; 2 Department of Gastroenterology and Hepatology, Nagasaki University Graduate School of Biomedical Sciences, 1-7-1 Sakamoto, Nagasaki City, Nagasaki 852-8501 Japan; 3 Department of Experimental Therapeutics, National Cancer Center Hospital East, 6-5-1 Kashiwanoha, Kashiwa, Chiba 277-8577 Japan; 4 Department of Pathology and Clinical Laboratories, National Cancer Center Hospital East, 6-5-1 Kashiwanoha, Kashiwa, Chiba 277-8577 Japan; 5 Division of Pathology, Exploratory Oncology Research & Clinical Trial Center, National Cancer Center, Kashiwa, Chiba 277-8577 Japan; 6 Department of Diagnostic Radiology, National Cancer Center Hospital East, 6-5-1 Kashiwanoha, Kashiwa, Chiba 277-8577 Japan; 7 Translational Research Support Section, National Cancer Center Hospital East, 6-5-1 Kashiwanoha, Kashiwa, Chiba 277-8577 Japan

**Keywords:** Gastric cancer, Trastuzumab deruxtecan, Interstitial lung disease, Optimize dosing, Tumor burden

## Abstract

**Background:**

Trastuzumab deruxtecan (T-DXd) is an antibody–drug conjugate targeting HER2-positive gastric cancer or gastroesophageal junction cancer (GC/GEJC). Although effective, T-DXd has notable toxicities, including interstitial lung disease (ILD). This study evaluated the efficacy, safety, and prognostic factors associated with T-DXd for GC/GEJC.

**Methods:**

A retrospective observational study was conducted at our institution by reviewing medical records of patients treated with T-DXd until September 2023. Eligible patients had unresectable advanced or recurrent GC/GEJC, HER2 status of IHC 3 + or IHC 2 + /ISH-positive, and prior treatment with trastuzumab-containing regimen.

**Results:**

Among the 101 patients analyzed, the initial T-DXd dose was 6.4 mg/kg in 77 patients and 5.4 mg/kg in 24 patients. The objective response rate was 54.3%, with a median PFS of 5.4 months and a median OS of 11.4 months. The significant prognostic factors for shorter PFS and OS included ECOG PS ≥ 1, presence of primary lesion, and peritoneal metastasis but not the initial T-DXd dose. ILD occurred in 14.9% of patients. Notably, higher T-DXd dose and smaller tumor burden were associated with a higher incidence of ILD.

**Conclusions:**

Several factors were associated with prognosis after T-DXd treatment in patients with GC/GEJC. Tumor burden is a potential risk factor for T-DXd-related ILD. Further studies are needed to optimize dosing based on tumor burden and to improve the therapeutic index.

**Supplementary Information:**

The online version contains supplementary material available at 10.1007/s10120-024-01560-z.

## Introduction

Trastuzumab deruxtecan (T-DXd) is an antibody–drug conjugate (ADC) that integrates an antibody against human epidermal growth factor receptor 2 (HER2) with a topoisomerase I inhibitor. It specifically binds to HER2-expressing tumor cells, internalized, and subsequently releases the cytotoxic payload to exert antineoplastic effects through inhibition of topoisomerase I [1]. The randomized phase II DESTINY-Gastric01 trial in Asia demonstrated that T-DXd as third- or later-line therapy significantly improved objective response rate (ORR) and overall survival (OS), compared with those of the physician’s choice of chemotherapy, among previously treated patients with HER2-positive gastric cancer or gastroesophageal junction cancer (GC/GEJC) [2]. Subsequently, the single-arm phase II DESTINY-Gastric02 trial confirmed the safety and efficacy of T-DXd in the US and Europe for patients with HER2-positive GC/GEJC after a first-line trastuzumab-containing regimen [3]. Based on these results, T-DXd (6.4 mg/kg) has become one of the standard therapeutic options for previously treated patients with HER2-positive advanced GC/GEJC [4–7].

However, gastrointestinal and hematological toxicities occurred in > 60% of patients treated with 6.4 mg/kg of T-DXd, leading to dose reduction in approximately 35% of patients during the treatment course [8]. Interstitial lung disease (ILD) has been identified as a notable and potentially life-threatening toxicity of T-DXd [9]. Therefore, identifying factors related with efficacy or ILD development will provide valuable information for the use of T-DXd in daily clinical practice. This study investigated the clinicopathological factors related with the efficacy and safety of T-DXd, with special attention to ILD, and suggested the appropriate management of T-DXd as third- or later-line treatment for patients with HER2-positive GC/GEJC.

## Materials and methods

### Study population

This retrospective observational study was conducted at the National Cancer Hospital East, Kashiwa, Japan. The medical records of patients who were treated with T-DXd at our institute until September 2023 were reviewed. The patients who met the following inclusion criteria were eligible for this study: (i) histologically confirmed unresectable advanced or recurrent GC/GEJC, (ii) HER2 status of immunohistochemistry (IHC) 3 + or IHC 2 + with positive in situ hybridization (ISH), (iii) received T-DXd monotherapy as third- or later-line, (iv) previously treated with trastuzumab-containing regimen, and (v) provided written informed consent to receive the treatment. HER2 status was locally assessed by two board-certified pathologists (NS and TK) using a monoclonal anti-HER2 antibody (PATHWAY HER2 [4B5], Ventana, Tucson, AZ) and fluorescence in situ hybridization using the PathVysion HER-2 Probe Kit (Abbott Laboratories, Abbott Park, IL). The dose of T-DXd was determined upon the investigator’s discretion and administered every 3 weeks as one cycle. Patients who were started on < 5.4 mg/kg of T-DXd were excluded from the analysis.

### Assessments

We collected clinicopathological data, including age, sex, Eastern Cooperative Oncology Group (ECOG) performance status (PS), disease status (metastatic or recurrent), HER2 status (3 + or 2 + /ISH-positive), primary tumor location (GEJ or gastric), presence of a primary tumor, metastatic sites, number of metastatic sites, and initial T-DXd dose. Tumor burden was estimated based on the following three variables: (a) cumulative diameter of the target lesions based on the Response Evaluation Criteria in Solid Tumors (RECIST) version 1.1 criteria, (b) cumulative diameter of all measurable lesions, without limitations on the number of lesions, and (c) number of measurable lesions, based on the assessment of two investigators (AJ and IN).

Among patients with measurable lesions, tumor response was evaluated based on the RECIST ver1.1. Progression-free survival (PFS) was defined as the interval between the start date of T-DXd and either progression or death from any cause, whichever occurred earlier. OS was defined as the interval between the start date of T-DXd and death. We assessed the grade of adverse events (AEs) using the Common Terminology Criteria for Adverse Events version 5.0. ILD was primarily diagnosed based on the clinical and radiological judgment of the treating physicians. In this study, a board-certified radiologist (SO) performed a retrospective review to confirm the initial diagnosis of ILD.

### Statistical analysis

Categorical variables were compared using chi-square test, whereas continuous variables were compared using two-sample t-test and the Mann–Whitney U test. Time-to-event analyses for PFS and OS were performed using Kaplan–Meier curves. Univariate analysis was conducted using Cox proportional hazards regression model, which included potential confounders, such as age, sex, ECOG PS, disease status, HER2 status, primary tumor location, presence of a primary tumor, metastatic site, number of metastatic sites, and initial T-DXd dose. Variables with a *P* value of < 0.05 in the univariate analysis were included in the subsequent multivariate analysis.

Furthermore, the same clinical characteristics used in the time-to-event analyses were compared based on the presence or absence of ILD, grade ≥ 3 neutropenia, and grade ≥ 2 decreased appetite. In addition, tumor burden was evaluated using the number and total tumor diameter of measurable lesions. Total tumor diameter was analyzed as a continuous variable using the Mann–Whitney U test and was also categorized into groups based on the median and tertile values for further analysis. Multivariate analysis using logistic regression was performed using the factors with a *P* value of < 0.05 in the group comparisons and previously reported risk factors for ILD (i.e., age and renal function).

A two-sided *P* value of < 0.05 was considered statistically significant. All analyses were performed using Python 3.8.5 within the Anaconda Navigator environment (version 1.9.12). The following Python libraries were utilized for data manipulation, analysis, and visualization: NumPy (version 1.19.2), Pandas (version 1.1.3), SciPy (version 1.5.2), Statsmodels (version 0.12.0), Lifelines (version 0.25.6), and Matplotlib (version 3.3.2).

## Results

### Patient characteristics

During the study period, 108 consecutive patients received T-DXd. After excluding 7 patients who were started on T-DXd at doses other than 5.4 or 6.4 mg/kg (Supplementary Fig. 1), 101 patients were enrolled in this study. The patient characteristics are listed in Table 1. The median age was 70 years (range, 30–85 years). The ECOG PS was 0 in 63 patients (62.4%) and ≥ 1 in 37.6%. HER2 IHC3 + was observed in 88 patients (87.1%). The median number of metastatic sites was two; the most common site of metastasis was the liver in 54 patients (53.5%), followed by the peritoneum in 39 patients (38.6%). Our study cohort did not include patients with history of ILD. In our cohort, the starting dose of T-DXd was 6.4 mg/kg in 77 patients (76.2%) and 5.4 mg/kg in 24 (23.8%) patients; compared with the former, the latter were generally older (median age, 69 years vs. 75 years) and comprised a higher proportion of patients with ECOG PS 1 or 2 (29.9% vs. 62.5%).

**Table 1:** Baseline patient characteristics

Characteristics	All (n = 101, %)	6.4 mg/kg (n = 77, %)	5.4 mg/kg (n = 24, %)	*P* value
Age				
Median (range)	70 (30–85)	69 (30–80)	75 (33–85)	0.013
≥ 65	65 (64.3)	48 (62.3)	17 (70.8)	0.61
≥ 75	27 (26.7)	13 (16.9)	14 (58.3)	< 0.001
Sex				
Male	77 (76.2)	60 (77.9)	17 (70.8)	0.53
Female	24 (23.7)	17 (22.1)	7 (29.2)	
ECOG PS				
0	63 (62.4)	54 (70.1)	9 (37.5)	0.008
1–2	38 (37.6)	23 (29.9)	15 (62.5)	
Disease status				
Metastatic	85 (84.2)	65 (84.4)	20 (83.3)	1.00
Recurrent	16 (15.8)	12 (15.6)	4 (16.7)	
HER2 IHC				
3 +	88 (87.1)	66 (85.7)	22 (91.7)	0.78
2 +	12 (11.9)	10 (13.0)	2 (8.3)	
Tumor location				
GEJ	22 (21.8)	13 (16.9)	9 (37.5)	0.06
Gastric	79 (78.2)	64 (83.1)	15 (62.5)	
Primary tumor				
Present	72 (71.3)	55 (71.4)	17 (70.8)	1.00
Absent	29 (28.7)	22 (28.6)	7 (29.2)	
Metastatic site				
Lymph node	75 (74.3)	60 (77.9)	15 (62.5)	0.37
Liver	54 (53.5)	42 (54.5)	12 (50.0)	0.40
Peritoneal	39 (38.6)	31 (40.3)	8 (33.3)	0.71
Lung	17 (16.8)	12 (15.6)	5 (20.8)	0.77
Bone	9 (8.9)	8 (10.4)	1 (4.2)	0.57
No of metastatic site				
< 2	35 (34.7)	24 (31.2)	11 (45.8)	0.28
≥ 2	66 (65.3)	53 (68.8)	13 (54.2)	

*ECOG PS* Eastern cooperative oncology group performance status; *ISH* in situ hybridization; *T-mab* trastuzumab; *GEJ* gastroesophageal junction

### Efficacy

**Fig. 1:**
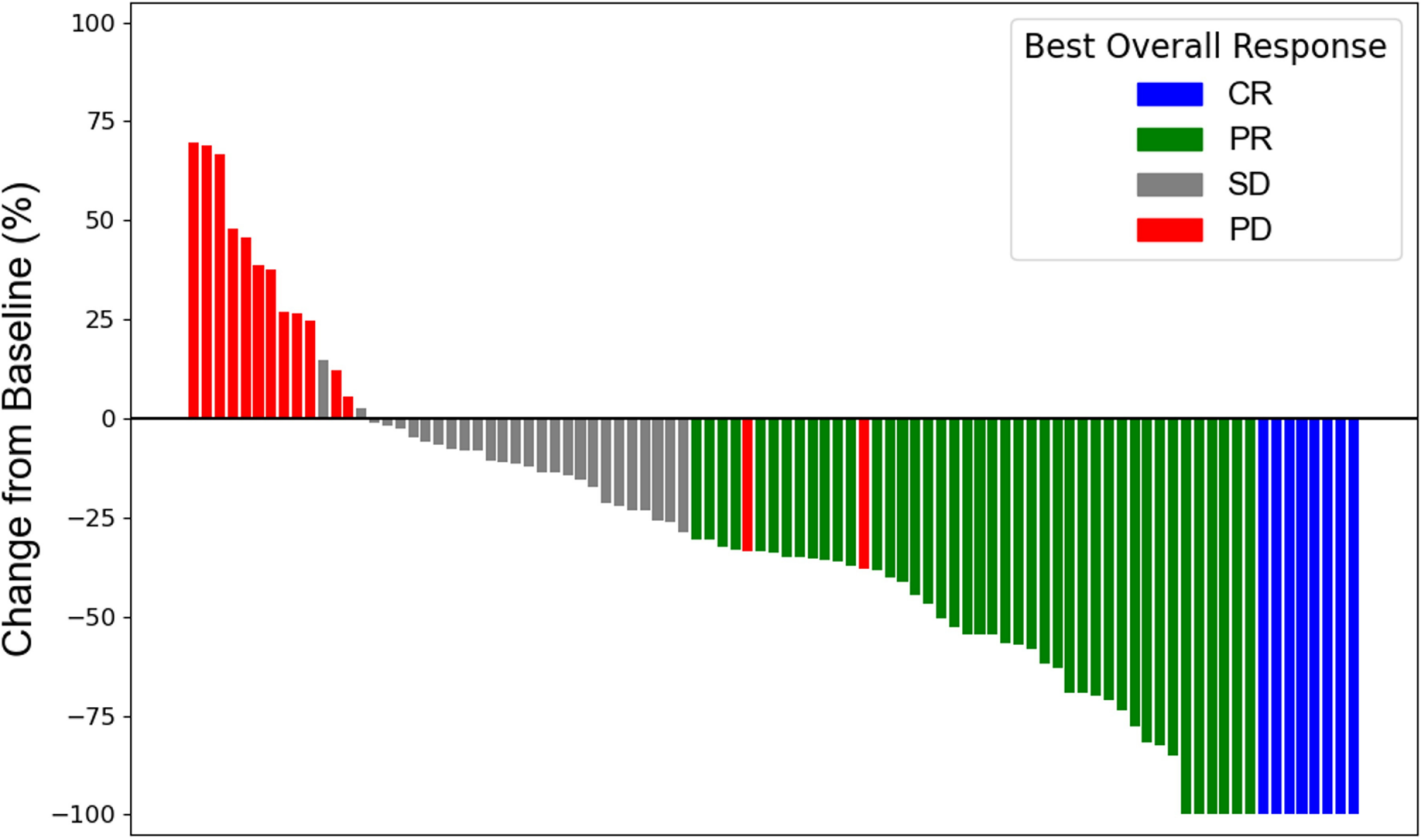
Best % change from baseline in the cumulative diameter of the target lesions. The waterfall plot displays each patient’s best response to T-DXd treatment compared with baseline, as measured by the maximum % reduction or minimum % increase in the cumulative of diameter of the target lesions. Among the 91 patients with at least one postbaseline tumor assessment, the best overall responses are indicated by the following colors: blue for CR, green for PR, gray for SD, and red for PD

The median number of treatment cycles was 6 (range, 1–70). At the time of data collection cutoff on June 1, 2024, with minimum follow-up duration of 9 months, 99 patients discontinued T-DXd mainly because of disease progression (n = 76, 76.8%); treatment-related AEs (n = 10, 10.1%); death from other causes (n = 2, 2.0%); completion of treatment after achieving complete response (CR) (n = 3, 3.0%); or other reasons (n = 8, 8.1%). Among patients with measurable lesions (n = 92), the ORR was 54.3% (n = 50), with 8 patients (8.7%) achieving CR and 42 patients (45.7%) partial response (PR). Additionally, 27 patients (29.3%) had stable disease (SD), and 14 patients (15.2%) experienced progressive disease (PD). One patient could not undergo postbaseline tumor assessment due to early death following treatment initiation. The waterfall plot of the maximum tumor shrinkage among the measurable lesions is presented in Fig. 1. Most patients (84.8%) had reduction in tumor size, with a median tumor reduction rate of 34.9%.

HER2 status immediately before T-DXd administration were assessable in 33 patients and had converted to negative in 13 patients (39.4%). Among 29 patients with measurable lesions, ORR was numerically lower in patients who were HER2-negative immediately before T-DXd than in those who maintained HER2 positivity (30.8% vs. 56.3%, P = 0.32). Disease progression and death events were observed in 91 (90.1%) and 80 (79.2%) patients, respectively. The median PFS and OS were 5.4 months [95% confidence interval (CI) 4.2–7.4] and 11.4 months (95% CI 8.1–13.8), respectively (Supplementary Fig. 2). Among 24 patients who were treated with an initial dose of 5.4 mg/kg, the ORR was 45.0% (9 of 20), the median PFS was 4.5 months, and the median OS was 11.5 months.

### Association with clinicopathological factors and clinical outcomes

The results of univariate and multivariate analyses are summarized in Tables 2, 3. Univariate analysis identified PS ≥ 1, presence of primary tumor, liver metastasis, peritoneal metastasis, and multiple metastatic sites to be significantly associated with shorter PFS. Multivariate analysis revealed presence of a primary tumor and peritoneal metastasis as the independent predictors of shorter PFS. For OS, univariate analysis identified PS ≥ 1, presence of a primary tumor, peritoneal metastasis, and multiple metastatic sites as significant factors, and multivariate analysis confirmed PS ≥ 1, presence of a primary tumor, and peritoneal metastasis as independent poor prognostic factors.

**Table 2:** Cox proportional hazard analysis for PFS in patients treated with T-DXd

Variables (PFS)	Univariate analysis			Multivariate analysis		
	HR	95% CI	*P* value	HR	95% CI	*P* value
Age, years; ≥ 65 vs < 65	1.19	0.77–1.85	0.43			
≥ 75 vs < 75	1.11	0.69–1.77	0.66			
Sex, Male vs Female	0.95	0.59–1.55	0.85			
ECOG PS, 1–2 vs 0	1.71	1.10–2.66	0.02	1.50	0.95–2.38	0.08
Disease status, Metastatic vs Recurrent	1.92	1.06–3.48	0.03	0.95	0.43–2.08	0.90
Renal dysfunction, Ccr; ≥ 60 vs < 60	0.99	0.64–1.56	0.98			
HER2-IHC, 3 + vs 2 +	1.15	0.61–2.17	0.66			
Tumor location, GEJ vs Gastric	1.18	0.72–1.96	0.50			
Primary tumor, Present vs Absent	2.46	1.50–4.04	< 0.001	2.52	1.25–5.07	0.010
Lymph node, Yes vs No	1.04	0.65–1.67	0.87			
Liver metastasis, Yes vs No	1.62	1.05–2.48	0.03	0.94	0.57–1.57	0.82
Peritoneal metastasis, Yes vs No	1.62	1.06–2.49	0.03	1.80	1.12–2.89	0.01
Lung metastasis, Yes vs No	0.92	0.53–1.61	0.77			
Bone metastasis, Yes vs No	1.24	0.62–2.48	0.54			
No of metastatic site, ≥ 2 vs < 2	1.56	1.00–2.45	0.05	1.14	0.69–1.89	0.62

*ECOG PS* Eastern cooperative oncology group performance status, *Ccr* creatinine clearance, *IHC* immunohistochemistry, *GEJC* gastroesophageal junction, *HR* hazard ratio, CI confidence interval

**Table 3:** Cox proportional hazard analysis for OS in patients treated with T-DXd

Variables (OS)	Univariate analysis			Multivariate analysis		
	HR	95% CI	*P* value	HR	95% CI	*P* value
Age, years; ≥ 65 vs < 65	1.25	0.78–2.01	0.36			
≥ 75 vs < 75	1.44	0.88–2.37	0.14			
Sex, Male vs Female	0.98	0.59–1.65	0.96			
ECOG PS, 1–2 vs 0	2.10	1.30–3.40	0.002	1.93	1.17–3.18	0.010
Disease status, Metastatic vs Recurrent	2.09	1.07–4.07	0.03	1.00	0.43–2.32	1.00
Renal dysfunction, Ccr; ≥ 60 vs < 60	0.98	0.61–1.58	0.93			
HER2–IHC, 3 + vs 2 +	0.83	0.44–1.58	0.58			
Tumor location, GEJ vs Gastric	0.86	0.49–1.53	0.61			
Primary tumor, Present vs Absent	2.49	1.46–4.23	< 0.001	2.60	1.29–5.27	0.008
Lymph node, Yes vs No	1.12	0.66–1.91	0.67			
Liver metastasis, Yes vs No	1.30	0.83–2.04	0.25			
Peritoneal metastasis, Yes vs No	2.04	1.29–3.20	0.002	2.52	1.52–4.16	< 0.001
Lung metastasis, Yes vs No	1.03	0.58–1.85	0.91			
Bone metastasis, Yes vs No	1.57	0.78–3.17	0.2			
No of metastatic site, ≥ 2 vs < 2	1.78	1.08–2.92	0.02	1.09	0.63–1.88	0.75
Initial dose, 6.4 mg/kg vs 5.4 mg/kg	0.80	0.47–1.36	0.42			

*ECOG PS* Eastern cooperative oncology group performance status, *Ccr* creatinine clearance, *IHC* immunohistochemistry, *GEJC* gastroesophageal junction, *HR* hazard ratio, *CI* confidence interval

### Safety

The AEs are listed in Supplementary Table 1. Almost all patients (n = 100, 99.0%) experienced at least one any grade AE during treatment. The most frequently observed nonhematological AEs were increased liver transaminase in 66 patients (65.3%), decreased appetite in 63 patients (62.3%), fatigue in 34 patients (33.7%), and nausea in 33 patients (32.7%). Grade 3 or 4 neutropenia and anemia were observed in 29 (28.7%) and 26 (25.7%) patients, respectively. The incidence of grade 3 or higher neutropenia tended to be 10% lower among patients who received an initial dose of 5.4 mg/kg than those who received 6.4 mg/kg. There were no differences in the other AEs between the two doses. (Supplementary Table 2).

### ILD and associated risk factors: a special interest

**Fig. 2:**
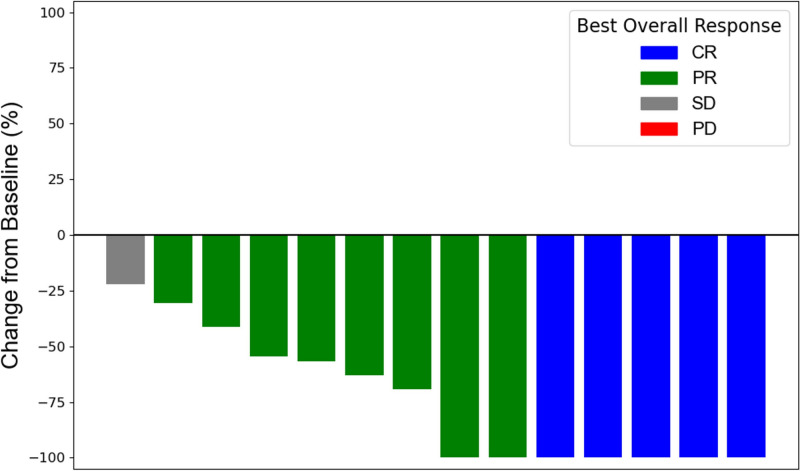
Maximum % change in target lesion size from baseline among patients who developed ILD. The waterfall plot shows the best response to treatment for the subset of 14 patients who developed ILD, as assessed by the greatest % decrease or smallest % increase in the cumulative diameter of the target lesions relative to baseline measurements. Each bar corresponds to an individual patient, with colors denoting their best overall response, as follows: blue for CR, green for PR, gray for SD, and red for PD

ILD was observed in 15 patients (14.9%). The onset of ILD varied, as follows: during cycles 1 to 3 (n = 3), cycles 4 to 6 (n = 4), cycles 7 to 9 (n = 4), and after cycles 10 (n = 6). ILD was the most common cause of T-DXd discontinuation (n = 9). Most cases of ILD were grade 1 (n = 6, 40.0%) or grade 2 (n = 8, 53.3%); grade 3 was observed in 1 patient (6.7%). No fatal ILD occurred in our cohort. Some baseline characteristics were imbalanced between patients with ILD and those without ILD (Table 4). ILD was significantly more frequent in patients without a primary tumor than in those with a primary tumor [10 of 29 (34.5%) vs. 5 of 72 (6.9%), *P* = 0.001]. Among the 24 patients who were started on 5.4 mg/kg of T-DXd, only 1 (4.2%) developed ILD; this rate was lower than that of the patients who were started on 6.4 mg/kg (14 of 77, 18.2%). Of the patients who developed ILD, 14 had at least one measurable lesion; 5 patients achieved CR and 8 patients achieved PR. Notably, the ORR was significantly higher in patients with ILD than in those without ILD (92.9% vs. 47.4%, *P* = 0.004). Tumor shrinkage was observed in all 14 patients who had target lesions by RECIST version 1.1 criteria (Fig. 2).

**Table 4:** Clinical characteristics according to ILD occurrence

Characteristics	With ILD (n = 15, %)	Without ILD (n = 86, %)	*P* value
Age			
Median (range)	69 (46–79)	70 (30–85)	0.63
≥ 65	9 (60.0)	56 (65.1)	0.92
< 65	6 (40.0)	30 (34.9)	
≥ 75	3 (20.0)	24 (27.9)	0.75
< 75	12 (80.0)	62 (72.1)	
Sex			
Male	13 (86.7)	64 (74.4)	0.48
Female	2 (13.3)	22 (25.6)	
ECOG PS			
0	13 (86.7)	50 (58.1)	0.07
1–2	2 (13.3)	36 (41.9)	
Disease status			
Metastatic	10 (66.7)	75 (87.2)	0.10
Recurrent	5 (33.3)	11 (12.8)	
Renal dysfunction			
Ccr ≥ 60	10 (66.7)	60 (70.0)	0.43
Ccr < 60	5 (33.3)	26 (30.0)	
HER2 IHC			
3 +	11 (73.3)	77 (89.5)	0.14
2 +	4 (26.7)	8 (9.3)	
Tumor location			
GEJ	3 (20.0)	19 (22.1)	1.00
Gastric	12 (80.0)	67 (77.9)	
Primary tumor			
Present	5 (33.3)	67 (77.9)	0.001
Absent	10 (66.7)	19 (22.1)	
Lymph node metastasis			
Yes	11 (73.3)	64 (74.4)	1.00
No	4 (26.7)	22 (25.6)	
Liver metastasis			
Yes	5 (33.3)	49 (57.0)	0.16
No	10 (66.7)	37 (43.0)	
Peritoneal metastasis			
Yes	5 (33.3)	34 (44.7)	0.87
No	10 (66.7)	52 (68.4)	
Lung metastasis			
Yes	1 (6.7)	16 (18.6)	0.44
No	14 (93.3)	72 (81.4)	
Bone metastasis			
Yes	1 (6.7)	8 (9.3)	1.00
No	14 (93.3)	78 (90.7)	
No of metastatic site			
≥ 2	7 (46.7)	27 (31.4)	0.39
< 2	8 (53.3)	59 (68.6)	
Initial dose			
6.4 mg/mg	14 (93.3)	63 (73.3)	0.17
5.4 mg/kg	1 (6.7)	23 (26.7)	

*ILD* interstitial lung disease, *ECOG PS* Eastern cooperative oncology group performance status, *Ccr* creatinine clearance

**Fig. 3:**
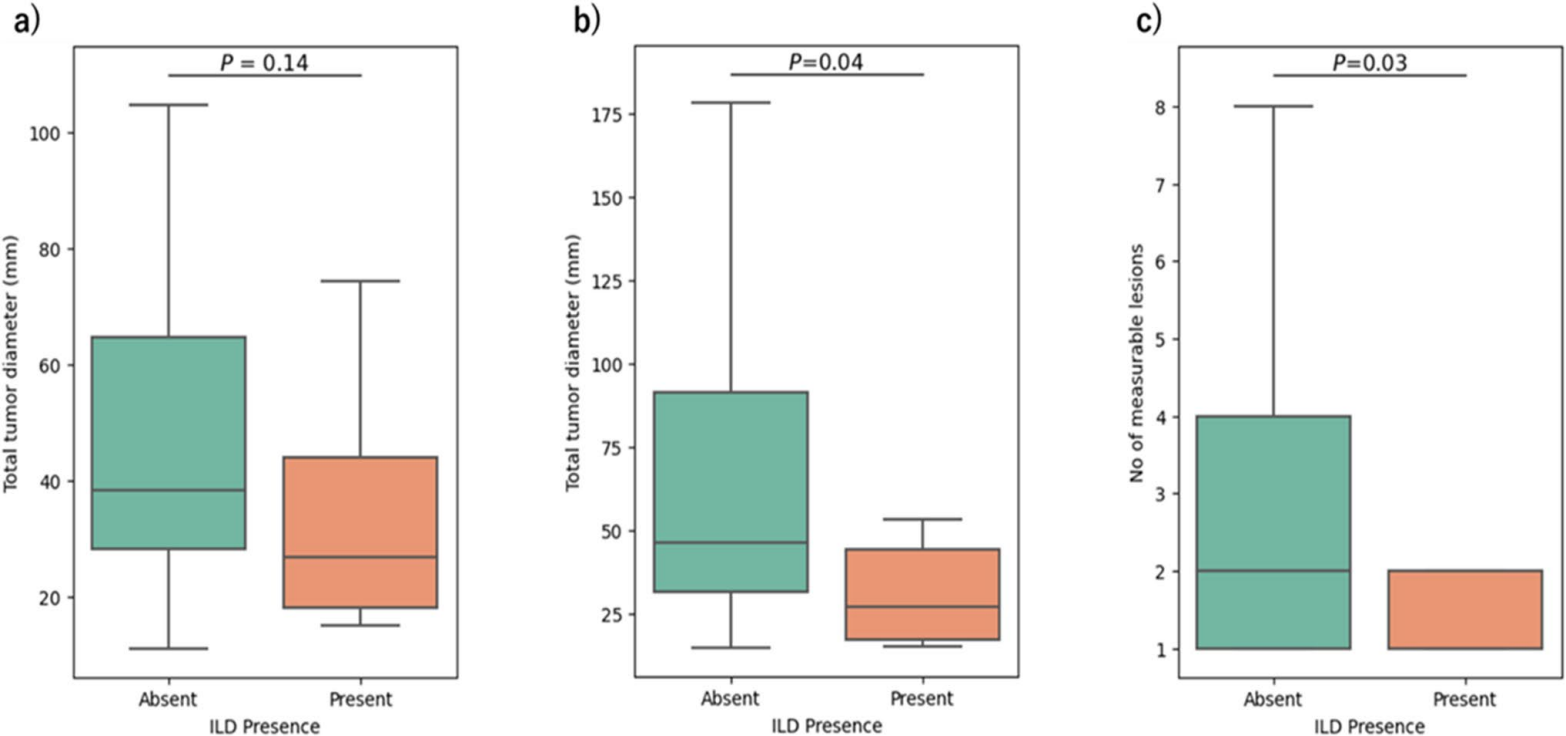
Relationship between ILD presence and tumor burden in patients who received 6.4 mg/kg T-DXd. Of the 72 patients who were treated with 6.4 mg/kg T-DXd and had one or more measurable lesions, 13 developed ILD. Tumor burden was assessed by the (**a**) total tumor diameter of the target lesions using the RECIST criteria (maximum of five lesions, up to two per organ); (**b**) total tumor diameter of all measurable lesions; and (**c**) number of measurable lesions

We evaluated the association between the incidence of ILD and tumor burden among 72 patients with measurable lesions and were treated with 6.4 mg/kg of T-DXd. Compared with patients without ILD, those with ILD tended to have smaller median total tumor diameter of the target lesions [26.9 mm (range, 15.0–38.9) vs. 38.3 mm (range, 11.1–171.7), *P* = 0.14]; significantly smaller median total diameter of all measurable lesions [26.9 mm (range, 15.0–152.1) vs. 46.3 mm (range, 14.7–399.3), *P* = 0.04]; and significantly lower median number of measurable lesions [1 (range, 1–5) vs. 2 (range, 1–18), P = 0.03] (Fig. 3).

Stratification of patients based on the lower tertile the total tumor diameter revealed significant differences in ILD occurrence. On multivariate analysis, the previously reported risk factors of age and renal function, as well as the presence or absence of primary tumors, were different between patients with ILD and those without ILD. In particular, patients without a primary tumor and those with total tumor diameter below the lower tertile remained independent predictors of ILD on multivariate analysis (Supplementary Tables 3a, b).

The other common T-DXd-associated AEs, such as grade 3 or higher neutropenia and grade 2 or higher decreased appetite, had no relationships with tumor burden (Supplementary Figs. 3a, b). Grade 3 or higher neutropenia was significantly more frequent in patients aged ≥ 65 years, whereas grade 2 or higher decreased appetite was more common in patients with multiple metastatic sites and peritoneal metastasis (Supplementary Tables 4a, b).

## Discussion

The clinical outcomes of T-DXd in this single-institute study were consistent with those reported in pivotal trials. Initiating T-DXd treatment at a reduced dose of 5.4 mg/kg achieved an ORR of 45.0% and was proven to be a safe option for vulnerable patients who were presumed to be intolerant to 6.4 mg/kg. To the best of our knowledge, this study was the first to demonstrate the relationship between the incidence of T-DXd-related ILD and tumor burden in patients with GC/GEJC.

We also assessed the relationships of various clinical factors with PFS and OS. The presence of peritoneal metastasis was significantly associated with shorter PFS and OS. A previous study reported that among 15 patients with HER2-positive primary lesions, a HER2-positive status was maintained in only 4 patients (26.7%) who developed peritoneal metastasis but was observed in 13 patients who developed liver metastases [10]. Although we did not assess the HER2 status in each metastatic site, a relatively low HER2 expression in peritoneal metastasis might have influenced the efficacy of T-DXd treatment. This result warrants further validation in larger cohorts. The ORR only tended to be higher in patients who maintained HER2 positivity than in those with HER2 loss, possibly due to the small sample size. The efficacy of T-DXd in patients who maintained HER2-positivity is under investigation in the ongoing randomized phase III DESTINY-Gastric 04 in a second-line setting [11].

Phase 1 and subsequent pivotal studies established the standard T-DXd dose as 6.4 mg/kg [2, 3, 12], which was higher than the dose for other indications because of different pharmacokinetic profiles [13]. However, in the DESINITY-GC01 study, dose reduction was required in 32% of patients [2]. In a postmarketing surveillance study in Japan (n = 1074), 20.6% were initially administered a reduced dose of T-DXd [14] based on investigator judgment. In our cohort, patients who were started on T-DXd at a dose of 5.4 mg/kg were more frail, compared with the patients treated with 6.4 mg/kg as the initial dose. Nevertheless, a 5.4 mg/kg initial dose achieved similar ORR, PFS, and OS with the 6.4 mg/kg initial dose in this study and compared favorably with other later-line treatment options [15–17]. These results suggested that dose reduction is appropriate in selected populations.

Similar to the reported incidence in a pivotal study [9], ILD was observed in 14.9% of patients and was the main reason for treatment discontinuation in this study. Therefore, early detection and careful management of ILD as a toxicity of T-DXd are needed. Notably, we observed a significant relationship between baseline tumor burden and ILD occurrence. The median total diameter of all measurable lesions was significantly smaller in patients with ILD than in those without ILD. Moreover, a total tumor diameter below the lower tertile was found to be an independent predictor of ILD, although exact cutoff value was difficult to determine because of the relatively small sample size. Furthermore, the significantly lower proportion of cases without a primary tumor in patients without ILD than in those with ILD implied that a small tumor burden may be predictive of ILD development.

The pathogenesis of ADC-induced ILD remains largely unknown, although ADC uptake through nontarget receptors is one hypothesis. In the study by Kumagai et al., administration of T-DXd or its unconjugated payload DXd in escalating doses to cynomolgus monkeys for over 3 months demonstrated dose-dependent ILD development in monkeys that were treated with T-DXd but not in those that received high doses of unconjugated DXd [18]. The previously reported high expression of Fcγ receptors and low HER2 expression in alveolar macrophages [19, 20] suggested that Fc-mediated nonspecific uptake may contribute to ADC-induced ILD. Similarly, a preclinical study on Trop2-eribulin ADC observed nonspecific ADC uptake by alveolar macrophages via Fcγ receptors, which released cytotoxic payload into lung tissue [21]. When the tumor burden is low, T-DXd tends to be relatively excessive, which may lead to its increased uptake by alveolar macrophages via Fcγ. The release of T-DXd payload within alveolar macrophages can cause accumulation of the highly lipophilic DXd in lung tissues, leading to alveolar damage. This could explain the high likelihood of developing ILD in patients with relatively low tumor burden. Supporting this hypothesis, all cases of ILD in this study showed tumor shrinkage, with a remarkable response rate of 92.7%; most of these cases were started on T-DXd without dose reduction. To improve the therapeutic index of ADCs, dose determination based on targeted expressing tumor volume warrant further evaluation.

During T-DXd treatment, the underlying mechanisms are believed to be different between ILD and hematological or gastrointestinal toxicities, such as neutropenia and decreased appetite. Our findings on the association between tumor burden and ILD onset supported the hypothesis that ILD is induced by a relative excess of ADC, leading to Fcγ-mediated uptake in lung tissue. On the other hand, there was no significant association between tumor burden and grade 3 or higher neutropenia or grade 2 or higher decreased appetite, which are likely caused by systemic exposure to DXd that is released in the bloodstream through cleavage of the linker from T-DXd. This difference in mechanisms is crucial for understanding the diverse AE profiles of T-DXd therapy and may have implications for patient monitoring and management strategies.

This study had several limitations. First, it was conducted at a single institution in Japan, which may limit the generalizability of the findings. Second, given the retrospective study design, all reported *P* values were nominal and should not be used to assert or cite statistical significance in a clinical setting. In addition, medical records review may not have comprehensively captured and potentially underestimated the incidence of nonhematologic AEs, such as gastrointestinal toxicity and fatigue. Furthermore, the total tumor diameter, which we used as an indicator of tumor burden, did not reflect the components of unmeasurable lesions, and the small sample size in certain subgroups makes interpretation of some results challenging and warrants caution when drawing conclusions.

## Conclusions

This study identified several prognostic factors associated with T-DXd treatment in patients with GC/GEJC. Initial dose reduction enabled T-DXd administration to a frail patient population. Tumor burden is a potential risk factor for T-DXd-related ILD. Further studies are needed to determine the optimal dosing based on tumor burden and to improve the clinical applicability of T-DXd for patients with advanced GC/GEJC.

## Supplementary Information

Below is the link to the electronic supplementary material.

Supplementary file1 (JPG 299 KB) Consort flow diagram. Of 108 patients with HER2-positive gastric or gastroesophageal junction cancer who received trastuzumab deruxtecan, 7 patients who received an initial dose of <5.4 mg/kg were excluded, leaving 101 patients eligible for the study

Supplementary file2 (JPG 531 KB) Kaplan–Meier analysis of PFS and OS. After a median follow-up of 38.3 months, **a** PFS was observed in 91 patients and had a median value of 5.4 months (95% CI 4.2–7.4) and **b** OS was observed in 80 patients and had a median value of 11.4 months (95% CI 8.1–13.8) for the entire cohort

Supplementary file3 (JPG 586 KB) Relationship between ≥grade 3 neutropenia and measurable lesions. Of the cohort of 72 patients treated with 6.4 mg/kg T-DXd who had one or more measurable lesions, 13 developed grade 3 or higher neutropenia. Tumor burden was assessed by the **a** total tumor diameter of target lesions per RECIST criteria (maximum five lesions, up to two per organ); **b** total tumor diameter of all measurable lesions; and **c** number of measurable lesions. Based on the results of the Mann–Whitney U test, patients with grade 3 or higher neutropenia and those without grade 3 or higher neutropenia had no significant differences in the **a** median total tumor diameter of the target lesions by RECIST [39.8 mm (range, 15.0–171.7 mm) vs. 35.5 mm (range, 14.7–147.0 mm), respectively, P = 0.19]; **b** median total diameter of all measurable lesions [39.8 mm (range, 15.0–398.9 mm) vs. 43.2 mm (range, 14.7–399.3 mm), respectively, P = 0.66]; and **c** median number of measurable lesions [1 (range, 1–16) vs. 2 (range, 1–18), respectively, P = 0.25]

Supplementary file4 (JPG 743 KB) Relationship between ≥grade 2 decreased appetite and measurable lesions. Of the cohort of 72 patients treated with 6.4 mg/kg T-DXd who had one or more measurable lesions, 13 developed grade 2 or higher decreased appetite. Tumor burden was assessed by the **a** total tumor diameter of target lesions per RECIST criteria (maximum of five lesions, up to two per organ); **b** total tumor diameter of all measurable lesions, and **c** number of measurable lesions. Based on the results of the Mann–Whitney U test, patients with grade 2 or higher decreased appetite and those without grade 2 or higher decreased appetite had no significant differences in the **a** median total tumor diameter of the target lesions by RECIST [39.1 mm (range, 15.0–147.0 mm) vs. 37.3 mm (range, 14.7–171.7 mm), respectively, P = 0.63]; **b** median total diameter of all measurable lesions [44.75 mm (range, 15.0–398.9 mm) vs. 39.7 mm (range, 14.7–399.3 mm), respectively, P = 0.47]; and **c** median number of measurable lesions [2 (range, 1–16] vs. 2 (range, 1–18), respectively, P = 0.29]

Supplementary file5 (DOCX 31 KB)
