# Supplementary figures and images for: Prognostic and predictive factors for the efficacy and safety of trastuzumab deruxtecan in HER2-positive gastric or gastroesophageal junction cancer
Source: Gastric Cancer. 2024 Nov 2;28(1):63–73. doi: 10.1007/s10120-024-01560-z (PMC11706866; doi:10.1007/s10120-024-01560-z)

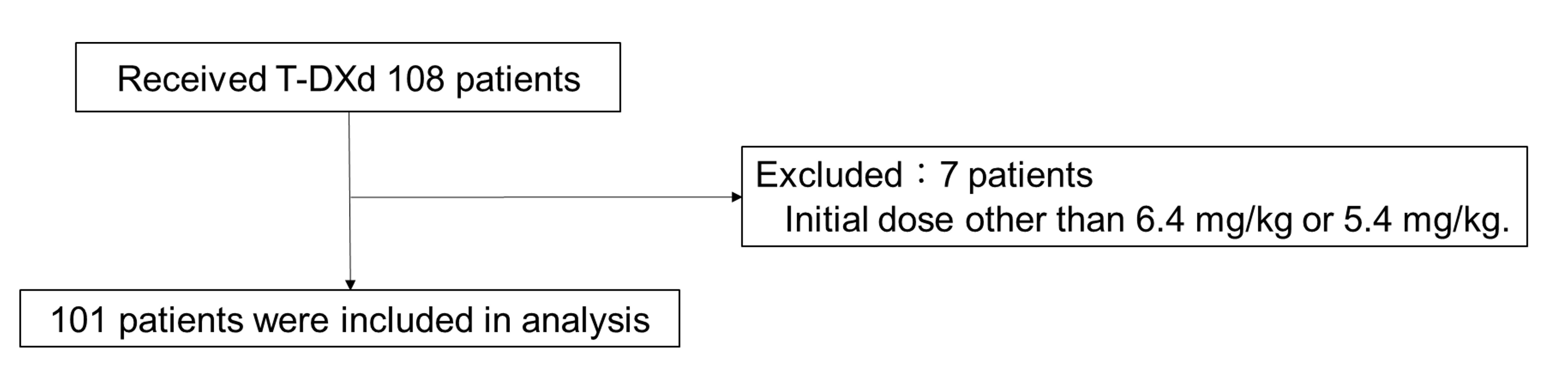

Supplement: Supplementary file 1 — Supplementary file1 (JPG 299 KB) Consort flow diagram. Of 108 patients with HER2-positive gastric or gastroesophageal junction cancer who received trastuzumab deruxtecan, 7 patients who received an initial dose of <5.4 mg/kg were excluded, leaving 101 patients eligible for the study [file 10120_2024_1560_MOESM1_ESM.jpg]

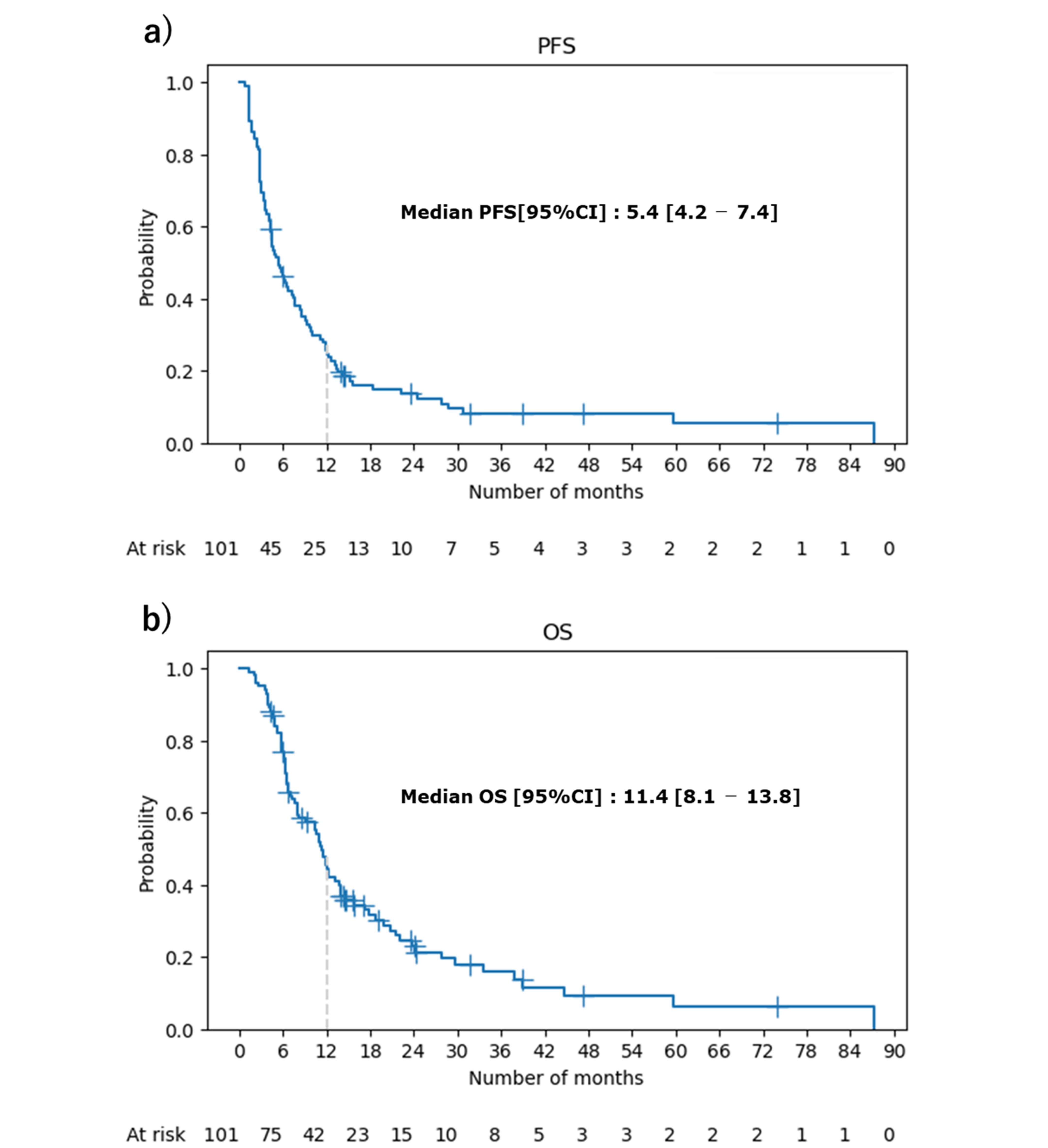

Supplement: Supplementary file 2 — Supplementary file2 (JPG 531 KB) Kaplan–Meier analysis of PFS and OS. After a median follow-up of 38.3 months, a PFS was observed in 91 patients and had a median value of 5.4 months (95% CI 4.2–7.4) and b OS was observed in 80 patients and had a median value of 11.4 months (95% CI 8.1–13.8) for the entire cohort [file 10120_2024_1560_MOESM2_ESM.jpg]

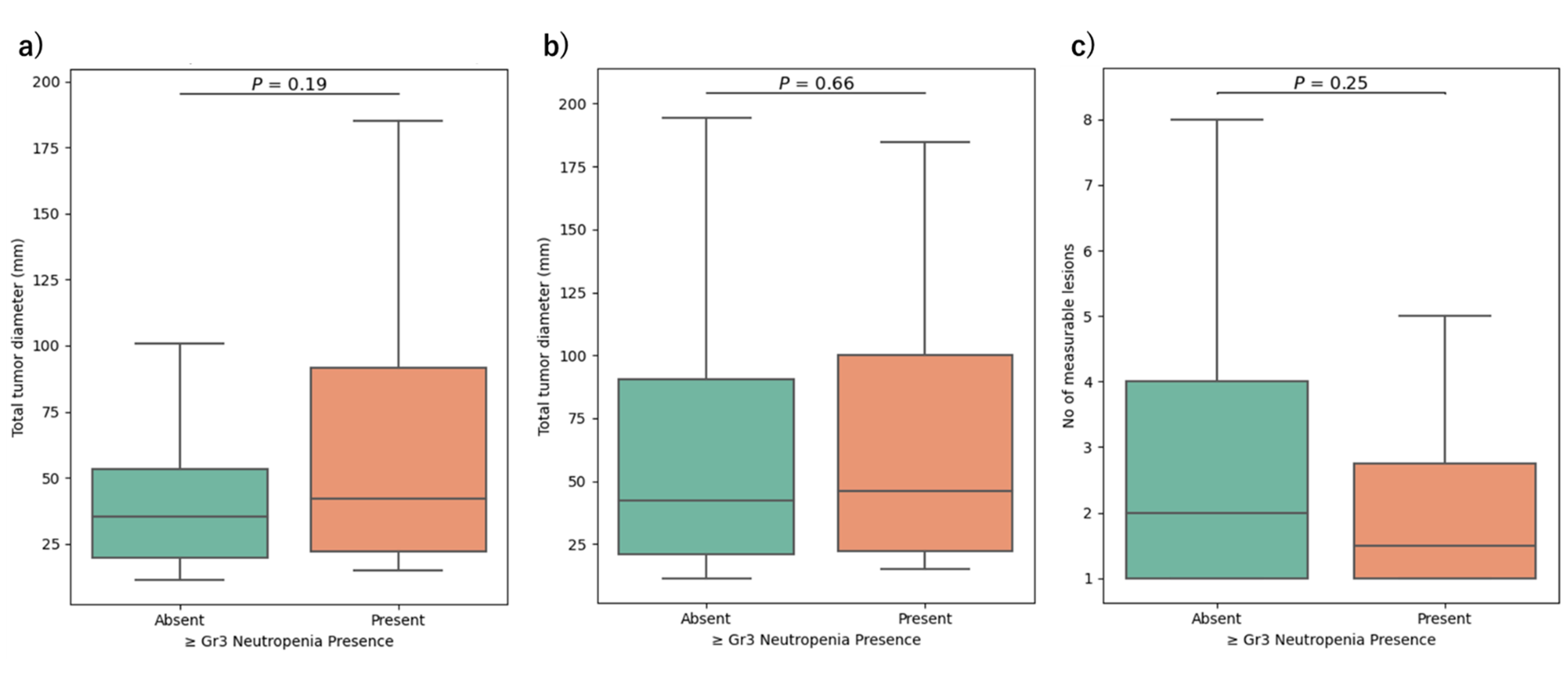

Supplement: Supplementary file 3 — Supplementary file3 (JPG 586 KB) Relationship between ≥grade 3 neutropenia and measurable lesions. Of the cohort of 72 patients treated with 6.4 mg/kg T-DXd who had one or more measurable lesions, 13 developed grade 3 or higher neutropenia. Tumor burden was assessed by the a total tumor diameter of target lesions per RECIST criteria (maximum five lesions, up to two per organ); b total tumor diameter of all measurable lesions; and c number of measurable lesions. Based on the results of the Mann–Whitney U test, patients with grade 3 or higher neutropenia and those without grade 3 or higher neutropenia had no significant differences in the a median total tumor diameter of the target lesions by RECIST [39.8 mm (range, 15.0–171.7 mm) vs. 35.5 mm (range, 14.7–147.0 mm), respectively, P = 0.19]; b median total diameter of all measurable lesions [39.8 mm (range, 15.0–398.9 mm) vs. 43.2 mm (range, 14.7–399.3 mm), respectively, P = 0.66]; and c median number of measurable lesions [1 (range, 1–16) vs. 2 (range, 1–18), respectively, P = 0.25] [file 10120_2024_1560_MOESM3_ESM.jpg]

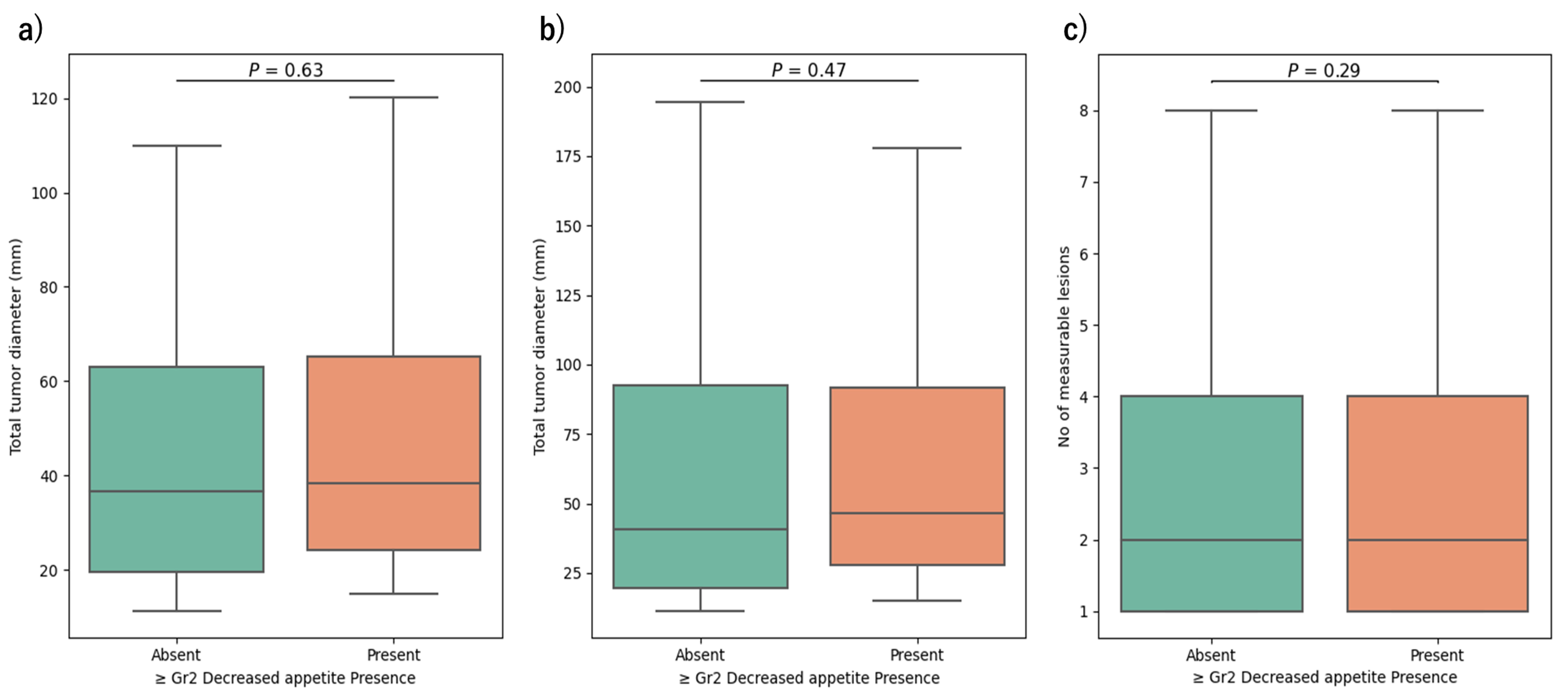

Supplement: Supplementary file 4 — Supplementary file4 (JPG 743 KB) Relationship between ≥grade 2 decreased appetite and measurable lesions. Of the cohort of 72 patients treated with 6.4 mg/kg T-DXd who had one or more measurable lesions, 13 developed grade 2 or higher decreased appetite. Tumor burden was assessed by the a total tumor diameter of target lesions per RECIST criteria (maximum of five lesions, up to two per organ); b total tumor diameter of all measurable lesions, and c number of measurable lesions. Based on the results of the Mann–Whitney U test, patients with grade 2 or higher decreased appetite and those without grade 2 or higher decreased appetite had no significant differences in the a median total tumor diameter of the target lesions by RECIST [39.1 mm (range, 15.0–147.0 mm) vs. 37.3 mm (range, 14.7–171.7 mm), respectively, P = 0.63]; b median total diameter of all measurable lesions [44.75 mm (range, 15.0–398.9 mm) vs. 39.7 mm (range, 14.7–399.3 mm), respectively, P = 0.47]; and c median number of measurable lesions [2 (range, 1–16] vs. 2 (range, 1–18), respectively, P = 0.29] [file 10120_2024_1560_MOESM4_ESM.jpg]
