# Supplementary material for: Prognostic and predictive factors for the efficacy and safety of trastuzumab deruxtecan in HER2-positive gastric or gastroesophageal junction cancer
Source: Gastric Cancer. 2024 Nov 2;28(1):63–73. doi: 10.1007/s10120-024-01560-z (PMC11706866; doi:10.1007/s10120-024-01560-z)
Supplement: Supplementary file 5 — Supplementary file5 (DOCX 31 KB) [file 10120_2024_1560_MOESM5_ESM.docx]

**Supplementary Table 1.** **Adverse Events**

| (n = 101, %) | Any Grade | ≥ Grade 3 |
| --- | --- | --- |
| **Hematological** |  |  |
| Neutropenia | 55 (54.5) | 29 (28.7) |
| Anemia | 92 (91.1) | 26 (25.7) |
| Thrombocytopenia | 18 (17.8) | 2 (2.0) |
| **Non–hematological** |  |  |
| Liver transaminase elevation | 66 (65.3) | 2 (2.0) |
| Decreased appetite | 63 (62.3) | 3 (3.0) |
| Fatigue | 34 (33.7) | 2 (2.0) |
| Nausea | 33 (32.7) | 0 (0.0) |
| ILD | 15 (14.9) | 1 (1.0) |
| Creatinine increased | 13 (12.9) | 0 (0.0) |
| Diarrhea | 11 (10.9) | 0 (0.0) |

Abbreviations: ILD, Interstitial Lung Disease;

**Supplementary Table 2.** **Adverse Events by Initial dose**

| Any Grade | 6.4mg/kg  (n = 77, %) | 5.4mg/kg  (n = 24, %) | *P* value |
| --- | --- | --- | --- |
| **Hematological** |  |  |  |
| Neutropenia | 44 (57.1) | 11 (45.8) | 0.46 |
| Anemia | 71 (92.2) | 21 (87.5) | 0.77 |
| Thrombocytopenia | 12 (15.6) | 6 (25.0) | 0.46 |
| **Non–hematological** |  |  |  |
| Liver transaminase elevation | 50 (64.9) | 16 (66.7) | 1.00 |
| Decreased appetite | 48 (62.3) | 15 (62.5) | 1.00 |
| Fatigue | 26 (33.8) | 8 (33.3) | 1.00 |
| Nausea | 27 (35.1) | 6 (25.0) | 0.45 |
| ILD | 14 (18.2) | 1 (4.1) | 0.17 |
| Creatinine increased | 9 (11.7) | 4 (16.7) | 0.77 |
| Diarrhea | 10 (13.0) | 1 (4.1) | 0.40 |
| ≥ Grade 3 |  |  |  |
| **Hematological** |  |  |  |
| Neutropenia | 24 (31.2) | 5 (20.8) | 0.47 |
| Anemia | 19 (24.7) | 7 (29.2) | 0.86 |
| Thrombocytopenia | 2 (2.6) | 0 (0.0) | 1.00 |
| **Non–hematological** |  |  |  |
| Liver transaminase elevation | 2 (2.6) | 0 (0.0) | 1.00 |
| Decreased appetite  Fatigue | 3 (3.9)  1 (1.3) | 0 (0.0)  0 (0.0) | 0.77 |
|  |  |  | 1.00 |
| Nausea | 0 (0.0) | 0 (0.0) | － |
| ILD | 1 (1.3) | 0 (0.0) | 1.00 |
| Creatinine increased | 0 (0.0) | 0 (0.0) | － |
| Diarrhea | 0 (0.0) | 0 (0.0) | － |

Abbreviations: ILD, Interstitial Lung Disease;

**Supplementary Table 3a.** **Among 72 patients with measurable lesions treated with 6.4 mg/kg of T-DXd, Clinical characteristics according to ILD occurrence**

| Characteristics | With ILD  (n = 13, %) | Without ILD  (n = 59,%) | *P* value |
| --- | --- | --- | --- |
| **Age** |  |  |  |
| ≥ 65 | 8 (61.5) | 38 (64.4) | 1.00 |
| < 65 | 5 (38.5) | 21 (35.6) |  |
| ≥ 75 | 2 (15.4) | 10 (16.9) | 1.00 |
| < 75 | 11 (84.6) | 49 (83.1) |  |
| **Sex** |  |  |  |
| Male | 12 (92.3) | 46 (78.0) | 0.43 |
| Female | 1 (7.7) | 13 (22.0) |  |
| **ECOG PS** |  |  |  |
| 0 | 11 (84.6) | 39 (66.1) | 0.33 |
| 1 – 2 | 2 (15.4) | 20 (33.9) |  |
| **Disease status** |  |  |  |
| Metastatic | 9 (69.2) | 53 (89.8) | 0.13 |
| Recurrent | 4 (30.8) | 6 (10.2) |  |
| **Renal Dysfunction** |  |  |  |
| Ccr ≥ 60 | 9 (69.2) | 45 (76.3) | 0.86 |
| Ccr < 60 | 4 (30.8) | 14 (23.7) |  |
| **HER2 IHC** |  |  |  |
| 3+ | 10 (76.9) | 51 (86.4) | 0.56 |
| 2+ | 3 (23.1) | 7 (11.9) |  |
| **Tumor location** |  |  |  |
| GEJ | 2 (15.4) | 10 (16.9) | 1.00 |
| Gastric | 11 (84.6) | 49 (83.1) |  |
| **Primary tumor** |  |  |  |
| Present | 5 (38.5) | 47 (80.0) | 0.008 |
| Absent | 8 (61.5) | 12 (20.0) |  |
| **Lymph node metastasis** |  |  |  |
| Yes | 9 (69.2) | 49 (83.1) | 0.09 |
| No | 4 (30.8) | 10 (116.9) |  |
| **Liver metastasis** |  |  |  |
| Yes | 5 (38.5) | 35 (59.3) | 0.29 |
| No | 8 (61.5) | 24 (40.7) |  |
| **Peritoneal metastasis** |  |  |  |
| Yes | 4 (30.8) | 23 (39.0) | 0.81 |
| No | 9 (69.2) | 36 (61.0) |  |
| **Lung metastasis** |  |  |  |
| Yes | 1 (7.7) | 9 (15.3) | 0.79 |
| No | 12 (92.3) | 50 (84.7) |  |
| **Bone metastasis** |  |  |  |
| Yes | 1 (7.7) | 6 (10.2) | 1.00 |
| No | 12 (92.3) | 53 (89.8) |  |
| **No of metastatic site** |  |  |  |
| ≥ 2 | 6 (46.2) | 44 (74.6) | 0.09 |
| < 2 | 7 (53.8) | 15 (25.4) |  |
| **Total tumor diameter*** |  |  |  |
| ≥ upper tertile (68.5mm) | 2 (15.4) | 22 (37.3) | 0.23 |
| < upper tertile | 11 (84.6) | 37 (62.7) |  |
| ≥ median (41.3mm) | 4 (30.8) | 32 (54.2) | 0.22 |
| < median | 9 (69.2) | 27 (45.8) |  |
| ≥ lower tertile (32.0mm) | 5 (38.5) | 43 (72.9) | 0.04 |
| < lower tertile | 8 (61.5) | 16 (27.1) |  |

Abbreviations: ILD, Interstitial Lung Disease; ECOG PS, Eastern Cooperative Oncology Group Performance Status; Ccr, Creatinine Clearance;

* Total tumor diameter of all measurable lesions

**Supplementary Table 3b.** **Logistic analysis for ILD occurrence in patients with measurable lesions treated with 6.4 mg/kg of T-DXd**

| Variables (ILD occurrence) | Univariate analysis | | | Multivariate analysis | | |
| --- | --- | --- | --- | --- | --- | --- |
|  | OR | 95% CI | *P* value | OR | 95% CI | *P* value |
| Age, years; ≥ 65 vs < 65 | 0.88 | 0.26 – 3.05 | 1.00 | 0.78 | 0.19 – 3.22 | 0.73 |
| ≥ 75 vs <75 | 0.89 | 0.17 – 4.65 | 1.00 |  |  |  |
| Sex, Male vs Female | 3.39 | 0.40 – 28.56 | 0.43 |  |  |  |
| ECOG PS, 0 vs 1 – 2 | 2.82 | 0.57 – 13.97 | 0.56 |  |  |  |
| Renal dysfunction, Ccr; < 60 vs ≥ 60 | 1.43 | 0.38 – 5.36 | 0.86 | 1.27 | 0.27 – 5.93 | 0.76 |
| HER2–IHC, 3+ vs 2+ | 0.46 | 0.10 – 2.08 | 0.56 |  |  |  |
| Tumor location, GEJC vs GC | 0.89 | 0.17 – 4.65 | 1.00 |  |  |  |
| Primary tumor, Absent vs Present | 6.27 | 1.73 – 22.64 | 0.008 | 6.31 | 1.61 – 24.67 | 0.008 |
| Lymph node metastasis, No vs + | 2.18 | 0.56 – 8,49 | 0.45 |  |  |  |
| Liver metastasis, No vs Yes | 2.33 | 0.68 – 8.00 | 0.29 |  |  |  |
| Peritoneal metastasis, No vs Yes | 1.43 | 0.40 – 5.22 | 0.81 |  |  |  |
| Lung metastasis, No vs Yes | 2.16 | 0.25 – 18.73 | 0.79 |  |  |  |
| Bone metastasis, No vs Yes | 1.36 | 0.15 – 12.36 | 1.00 |  |  |  |
| No of metastatic site, < 2 vs ≥ 2 | 3.42 | 0.99 – 11.80 | 0.09 |  |  |  |
| Total tumor diameter*, < upper tertile vs ≥ upper tertile | 3.27 | 0.66 – 16.14 | 0.23 |  |  |  |
| Total tumor diameter*, < median vs ≥ median | 2.67 | 0.74 – 9.63 | 0.22 |  |  |  |
| Total tumor diameter*, < lower tertile vs ≥ lower tertile | 4.30 | 1.22 – 15.10 | 0.04 | 4.44 | 1.14 – 17.31 | 0.03 |

Abbreviations: ILD, Interstitial Lung Disease; Ccr, Creatinine Clearance; OR, Odds Ratio; CI, Confidence Interval;

* Total tumor diameter of all measurable lesions

**Supplementary Table 4a.** **Clinical characteristics according to ≥ Grade3 Neutropenia occurrence**

| Characteristics | With ≥ Gr3 Neutropenia  (n = 29, %) | Without ≥ Gr3  Neutropenia  (n = 72,%) | *P* value |
| --- | --- | --- | --- |
| **Age** |  |  |  |
| Median (range) | 72 (49 – 83) | 69 (30 – 85) | 0.03 |
| ≥ 65 | 24 (82.8) | 41 (56.9) | 0.03 |
| < 65 | 5 (17.2) | 31 (43.1) |  |
| ≥ 75 | 12 (41.4) | 15 (20.8) | 0.06 |
| < 75 | 17 (58.6) | 57 (79.1) |  |
| **Sex** |  |  |  |
| Male | 24 (82.8) | 53 (73.6) | 0.47 |
| Female | 5 (17.2) | 19 (26.4) |  |
| **ECOG PS** |  |  |  |
| 0 | 19 (65.5) | 44 (61.1) | 0.85 |
| 1 – 2 | 10 (34.5) | 28 (38.9) |  |
| **Disease status** |  |  |  |
| Metastatic | 24 (82.8) | 61 (84.7) | 1.00 |
| Recurrent | 5 (17.2) | 11 (15.3) |  |
| **Renal Dysfunction** |  |  |  |
| Ccr ≥ 60 | 20 (69.0) | 50 (69.4) | 1.00 |
| Ccr < 60 | 9 (31.0) | 22 (30.6) |  |
| **HER2 IHC** |  |  |  |
| 3+ | 23 (79.3) | 65 (90.3) | 0.17 |
| 2+ | 6 (20.7) | 6 (8.3) |  |
| **Tumor location** |  |  |  |
| GEJ | 6 (20.7) | 16 (22.2) | 1.00 |
| Gastric | 23 (79.3) | 56 (77.8) |  |
| **Primary tumor** |  |  |  |
| Present | 19 (65.5) | 53 (73.6) | 0.57 |
| Absent | 10 (34.5) | 19 (26.4) |  |
| **Lymph node metastasis** |  |  |  |
| Yes | 20 (69.0) | 55 (76.3) | 0.60 |
| No | 9 (31.0) | 17 (23.7) |  |
| **Liver metastasis** |  |  |  |
| Yes | 15 (51.7) | 39 (54.2) | 0.99 |
| No | 14 (48.3) | 33 (45.8) |  |
| **Peritoneal metastasis** |  |  |  |
| Yes | 12 (41.4) | 27 (37.5) | 0.89 |
| No | 17 (58.6) | 45 (62.5) |  |
| **Lung metastasis** |  |  |  |
| Yes | 3 (10.3) | 14 (19.4) | 0.42 |
| No | 26 (89.7) | 58 (80.6) |  |
| **Bone metastasis** |  |  |  |
| Yes | 3 (10.3) | 6 (8.3) | 1.00 |
| No | 26 (89.7) | 66 (91.7) |  |
| **No of metastatic site** |  |  |  |
| ≥ 2 | 19 (65.5) | 47 (65.3) | 1.00 |
| < 2 | 10 (34.5) | 25 (34.7) |  |
| **Initial Dose** |  |  |  |
| 6.4mg/mg | 24 (82.8) | 53 (73.6) | 0.47 |
| 5.4mg/kg | 5 (17.2) | 19 (26.4) |  |

Abbreviations: ILD, Interstitial Lung Disease; ECOG PS, Eastern Cooperative Oncology Group Performance Status; Ccr, Creatinine Clearance;

**Supplementary Table 4b.** **Clinical characteristics according to ≥ Grade2 Decreased appetite**

| Characteristics | With ≥ Gr2 Decreased appetite  (n = 29, %) | Without ≥ Gr2 Decreased appetite  (n = 72,%) | *P* value |
| --- | --- | --- | --- |
| **Age** |  |  |  |
| Median (range) | 71 (37 – 83) | 69 (30 – 85) | 0.09 |
| ≥ 65 | 22 (75.9) | 43 (59.7) | 0.19 |
| < 65 | 7 (24.1) | 29 (40.3) |  |
| ≥ 75 | 11 (37.9) | 16 (22.2) | 0.17 |
| < 75 | 18 (62.1) | 56 (77.8) |  |
| **Sex** |  |  |  |
| Male | 25 (86.2) | 52 (72.2) | 0.22 |
| Female | 4 (13.8) | 20 (27.8) |  |
| **ECOG PS** |  |  |  |
| 0 | 15 (51.7) | 48 (66.7) | 0.61 |
| 1 – 2 | 14 (48.3) | 24 (33.3) |  |
| **Disease status** |  |  |  |
| Metastatic | 25 (86.2) | 60 (83.3) | 0.95 |
| Recurrent | 4 (13.8) | 12 (16.7) |  |
| **Renal Dysfunction** |  |  |  |
| Ccr ≥ 60 | 17 (58.6) | 53 (73.6) | 0.22 |
| Ccr < 60 | 12 (41.4) | 19 (26.4) |  |
| **HER2 IHC** |  |  |  |
| 3+ | 25 (86.2) | 63 (87.5) | 0.99 |
| 2+ | 4 (13.8) | 8 (11.1) |  |
| **Tumor location** |  |  |  |
| GEJ | 5 (17.2) | 17 (23.6) | 0.66 |
| Gastric | 24 (82.8) | 55 (76.4) |  |
| **Primary tumor** |  |  |  |
| Present | 22 (75.9) | 50 (69.4) | 0.69 |
| Absent | 7 (14.1) | 22 (30.6) |  |
| **Lymph node metastasis** |  |  |  |
| Yes | 21 (72.4) | 54 (75.0) | 0.99 |
| No | 8 (27.6) | 18 (25.0) |  |
| **Liver metastasis** |  |  |  |
| Yes | 15 (51.7) | 39 (54.2) | 0.99 |
| No | 14 (48.3) | 33 (45.8) |  |
| **Peritoneal metastasis** |  |  |  |
| Yes | 19 (65.5) | 20 (27.8) | <0.001 |
| No | 10 (34.5) | 52 (72.2) |  |
| **Lung metastasis** |  |  |  |
| Yes | 5 (17.2) | 12 (16.7) | 1.00 |
| No | 24 (82.8) | 60 (83.3) |  |
| **Bone metastasis** |  |  |  |
| Yes | 3 (10.3) | 6 (8.3) | 1.00 |
| No | 26 (89.7) | 66 (91.7) |  |
| **No of metastatic site** |  |  |  |
| ≥ 2 | 24 (82.8) | 42 (58.3) | 0.04 |
| < 2 | 5 (17.2) | 30 (41.7) |  |
| **Initial Dose** |  |  |  |
| 6.4mg/mg | 24 (82.8) | 53 (73.6) | 0.47 |
| 5.4mg/kg | 5 (17.2) | 19 (26.4) |  |

Abbreviations: ILD, Interstitial Lung Disease; ECOG PS, Eastern Cooperative Oncology Group Performance Status; Ccr, Creatinine Clearance;
